# Conduction system pacing using electro-anatomical mapping-guided system vs. fluoroscopy: a systematic review, meta-analysis and economic evaluation

**DOI:** 10.3389/fcvm.2024.1519127

**Published:** 2025-01-14

**Authors:** Beatriz León-Salas, Diego Infante-Ventura, Aránzazu Hernández-Yumar, Renata Linertová, Estefanía Herrera-Ramos, Alezandra Torres-Castaño, Himar González-Pacheco, Analía Abt-Sacks, Javier García-García, Raúl Quirós-López, María M. Trujillo-Martín

**Affiliations:** ^1^ Canary Islands Health Research Institute Foundation (FIISC), Santa Cruz de Tenerife, Spain; ^2^ Evaluation Unit (SESCS), Canary Islands Health Service (SCS), Santa Cruz de Tenerife, Spain; ^3^ Spanish Network of Agencies for Health Technology Assessment for the National Health Service (RedETS), Madrid, Spain; ^4^ Research Network on Chronicity, Primary Care, and Health Promotion (RICAPPS), Carlos III Health Institute (Instituto de Salud Carlos III), Madrid, Spain; ^5^ Quality and Patient Safety Unit, Nuestra Señora de Candelaria University Hospital, Santa Cruz de Tenerife, Spain; ^6^ Internal Medicine Unit, University Hospital Costa del Sol, Málaga, Spain

**Keywords:** bradycardias, conduction system pacing, electro-anatomical mapping, fluoroscopy, His bundle pacing, left bundle pacing, mapping system, artificial pacemaker

## Abstract

**Introduction:**

Electro-anatomical mapping (EAM) system has been shown as an alternative procedure to fluoroscopy for conduction system pacing (CSP) in patients with severe bradyarrhythmia, however its beneficial and harmful effects has not been assessed in a systematic review (SR). We sought to assess their safety, effectiveness and cost-effectiveness.

**Methods:**

A SR of the available scientific literature was conducted on the safety, effectiveness, and cost-effectiveness of CSP using EAM system versus fluoroscopy in patients with severe bradyarrhythmia. A partial economic evaluation was carried out to compare the costs of both strategies from the perspective of the Spanish National Health System. A budget impact analysis was also conducted with a five-year horizon.

**Results:**

Seven comparative observational studies (*N* = 231), analyzing the use of EAM versus fluoroscopy were selected. Statistically significant differences were observed in total fluoroscopy time: −9.87 minutes (95%CI:−14.20, −5.53, *p* < 0.01; *I*^2^ = 95%; *k* = 7; *n* = 231); His-lead fluoroscopic time: −8.08 minutes (95%CI:−10.36, −5.81, *p* < 0.01; *I*^2^ = 0%; *k* = 2; *n* = 50); and His-lead radiation dose: −17.21 mGy (95%CI:−24.08, −10.34, *p* < 0.01; *k* = 1; *n* = 20). No differences in total fluoroscopy dose, successful procedure or safety were found. The use of EAM represents an increase of EUR 1397.81 per patient and a net budget impact of EUR 1.63 million.

**Discussion:**

EAM is a valuable alternative for patients who should not be exposed to ionizing radiation with similar effectiveness and safety than fluoroscopy. However, the inclusion of EAM systems, for the indication under study, in routine clinical practice would mean an increase in costs for the Spanish National Health System.

**Systematic Review Registration:**

https://www.crd.york.ac.uk/PROSPERO/display_record.php?RecordID=421072, identifier (CRD42023421072).

## 1 Introduction

Bradycardias can significantly hinder proper cardiovascular function (1, 2). Within the complex landscape of bradycardias, symptomatic sinus node dysfunction (SND) and atrioventricular conduction blocks (AVB) are particularly prominent (3, 4).

Conduction System Pacing (CSP) has been a significant advance in pacemaker technology and has become a crucial component in modern cardiac care for treating bradyarrhythmias. The implantation of CSP has risen dramatically and is gaining mainstream acceptance across Europe (5). CSP, achieved through permanent His bundle pacing (HBP) or left bundle branch pacing (LBBP), is now established as an alternative physiological pacing option for patients with an indication for right ventricular pacing and cardiac resynchronization therapy (6–8).

CSP, targeting the stimulation of the bundle of His or the left conduction system, offers an alternative to conventional pacing by replicating ventricular activation and avoiding the ventricular asynchrony associated with traditional right ventricular pacing. However, locating the His bundle region and lead implantation is often technically challenging, time-consuming, fluoroscopy-intensive, and requires high precision to map for an appropriate pacing site compared to traditional right ventricular pacing (6). The higher fluoroscopic exposure can cause damage to both the patients and operators (9–12).

The use of the three-dimensional (3D) electro-anatomical mapping (EAM) systems to guide CSP lead implantation is increasingly being used to minimize or eliminate radiation doses (13–15). The European Heart Rhythm Association (EHRA) highlights the importance of EAM in complex anatomical cases, such as patients with left atrial area >40 cm², upgrade post-pacemaker-induced cardiomyopathy (PICM), upgrade post failed biventricular (BiV) implants, and congenital heart disease (CHD) (5). However, the studies included in this systematic review predominantly focused on CSP implantation in patients with standard anatomy. This study evaluates the EAM-guided CSP in this population.

The aim of this study was to evaluate the safety, clinical effectiveness, cost-effectiveness, and overall costs of using EAM compared to fluoroscopy in guiding the implantation of CSP for the treatment of bradyarrhythmia (SND and AVB).

## 2 Materials and methods

### 2.1 Systematic review on safety, effectiveness, and cost-effectiveness

A systematic review (SR) of the scientific literature was conducted according to the methodology developed by the Cochrane Collaboration (16), with reporting in accordance with the PRISMA (Preferred Reporting Items for Systematic reviews and Meta-Analyses) statement (17).

#### 2.1.1 Information sources and search strategy

The following electronic databases were searched (from database inception to May 2023): MEDLINE (OVID), Embase (Elsevier), the Cochrane Central Register of Controlled Trials (Wiley) and CINAHL (EBSCOhost). The search strategy was initially developed for MEDLINE and then adapted for each of the other databases. It included both controlled vocabulary and text-word terms, and also commercial names of devices related to CSP. No time or language limits were imposed. The search strategies are available in Supplementary Table S1.

Finally, the reference lists of all pertinent papers were examined to identify additional studies that could meet the selection criteria but were not retrieved through the electronic search in biomedical databases.

#### 2.1.2 Selection criteria

Studies were eligible for inclusion if they fulfilled the following criteria:

a. Type of study: Randomized controlled trials (RCTs) and full economic evaluations (EE) were included. If RCTs were not available, non-randomized controlled trials (nRCTs) were considered. In the absence of these, observational studies with a control group were considered. Depending on the quality and quantity of the EE identified, cost-consequences analysis and partial economic evaluations for Spain were also considered for inclusion.
b. Population: Patients with bradyarrhythmias requiring permanent pacemakers (symptomatic SND or AVB). We included studies with mixed populations [e.g., patients requiring pacemakers for bradyarrhythmias and those with cardiac resynchronization therapy (CRT) indications] only if the results for the sugroup meeting our inclusion criteria were reported separately, or if this subgroup represented approximately 80% of the study population.
c. Intervention: CSP (includes both HBP and LBBP) using EAM.
d. Comparator: CSP (both HBP and LBBP) using fluoroscopy.
e. Outcome measures: To be included, studies must report any of the following outcomes:
  i. Effectiveness and safety outcomes: success of the procedure, adverse effects (e.g., development of tumors, genetic defects, fetal or newborn malformations, spontaneous abortions, etc.), complications (e.g., periprocedure or postprocedure), total procedure time, total time and dose of fluoroscopy, device parameters (e.g., impedance, stimulation thresholds, R-wave, signal quality of intracavitary electrogram, QRS width), health-related quality of life (HRQoL), other patient reported outcomes (PROMs), and Patient Reported Experiences (PREMs).
  ii. Economic outcomes: incremental cost-effectiveness ratio (ICER), costs in monetary units, and benefits in quality-adjusted life years (QALYs), life years (LYs) gained, monetary units or in any safety or effectiveness outcomes.
f. Language: Spanish or English.
g. Publication type: Only full original publications.

#### 2.1.3 Study selection process

Two reviewers independently screened the titles and abstracts of the references identified by the electronic search. The full texts of studies meeting the predefined selection criteria were thoroughly examined and evaluated for inclusion. Any uncertainties or discrepancies between reviewers were resolved through discussion or consultation with a third reviewer, ultimately reaching a consensus.

#### 2.1.4 Data collection process and risk of bias assessment

Data extraction and the assessment of risk of bias were carried out independently and simultaneously by two reviewers. Any discrepancies were resolved through consultation with a third reviewer. An Excel-based data extraction form was prepared by the authors, subjected to pilot testing on two studies, and refined accordingly. The extracted data encompassed various aspects, including general study characteristics (e.g., first author, publication year, country, funding, and conflicts of interest), design and methodology details (e.g., objective, number of centers and duration of follow-up), sample characteristics (e.g., age, sex, and pathology), intervention and comparator details, outcomes, and results.

The Cochrane risk of bias tool for ECAs (RoB 2) was applied to evaluate the risk of bias in RCT (18). The Risk Of Bias In Non-randomised Studies—of Interventions (ROBINS-I) (19) and—of Exposures (ROBINS-E) (20) were used to assess the risk of bias in nRCT and observational studies.

The Drummond checklist (21) and the Critical appraisal tools (FLC 3.0) (22) for Spanish studies were used to evaluate the methodological quality of EE.

#### 2.1.5 Publication bias assessment

Following the Cochrane Collaboration recommendations (16), the presence of publication bias was assessed by computing the Egger's test, with the statistical significance level set at 0.05. The analysis used the metabias commands in the Stata Statistical Software (STATA 17, StataCorp. 2021. Stata Statistical Software: Release 17. College Station, TX: StataCorp LLC).

#### 2.1.6 Synthesis of the evidence

The quantitative synthesis of results was conducted through meta-analysis using the Review Manager software (RevMan, version 5.4.1. Copenhagen: The Nordic Cochrane Center, The Cochrane Collaboration, 2020). The Mantel-Haenszel method was applied to estimate the pooled risk ratio (RR) for each dichotomous variable, with continuity correction applied for studies with zero events in one or both groups, the generic inverse variance method was used, along with the mean difference (MD) or standardized mean difference (SMD) to combine continuous variables (23).

Heterogeneity was assessed using Higgins’ I² statistic. In cases of heterogeneity (*I*² ≥ 50% or *p* < 0.1), meta-analyses were conducted using a random-effects model. A sensitivity analysis was performed by systematically omitting each study individually to assess the stability of the overall effect estimate. In the absence of both clinical and statistical heterogeneity, a fixed-effect model was used. The pacing modality (HBP or LBBP) was extracted for each study to explore potential differences in outcomes, as HBP procedures are generally associated with longer procedural and fluoroscopy times compared to LBBP. When not explicitly reported, we inferred this information from the study descriptions.

#### 2.1.7 Certainty of evidence assessment

The certainty of evidence for all outcomes was assessed using the GRADE approach, which considered key outcomes across the following domains: risk of bias, inconsistency, indirectness, imprecision, and publication bias (24). The GRADEpro app was used to assign ratings to the evidence and generate the GRADE evidence profile. The certainty of evidence was categorized as high (indicating a high level of confidence that the true effect aligns closely with the estimated effect), moderate, low, or very low (indicating minimal confidence in the estimated effect).

### 2.2 Economic evaluation

Besides the SR, a cost analysis for Spain was conducted. The evaluated strategy involved guiding the CSP using an EAM, whereas the comparator used fluoroscopy, which is considered the standard clinical practice in Spain.

The target population was the same as described previously in the selection criteria. Based on data from the Spanish Pacemaker Registry (25), 38,893 pacemakers were implanted in Spain in 2021, of which 92% were first implants. Of these, 15.1% were due to SND and 13.7% were due to AVB (to perform this, the data for second degree AVB was taken), respectively. In addition, the Abbott company reported that around 2% of these implants were performed in the vulnerable population under study. Therefore, it was estimated that the target population is around 207 patients per year.

The perspective of the National Health Service was adopted, including only direct health costs. The time horizon considered was limited to the duration of the CSP implantation procedure, as the short-, medium- and long-term health impacts of the evaluated technology are unknown. Due to the short time horizon, no discounting of cost was applied. The cost per patient for each strategy and the incremental cost were estimated. The analyses were conducted using Microsoft Excel 2013, and methods and results are presented in accordance with CHEERS (Consolidated Health Economic Evaluation Reporting Standards) (26).

#### 2.2.1 Parameters

Data on resource use and costs were provided by the manufacturer Abbott Laboratories, based on Ensite X EAM.

In both alternatives, CSP implantation requires the same resources (pulse generator, introducers, electrodes, measurement cables, and implant tool) and equipment (ultrasound, catheter and polygraph). Therefore, the main difference between the alternatives lies in the use of the EAM vs. fluoroscopy. These common costs were not included in the analysis, as their consideration would not impact the incremental cost. The remaining costs are expressed in euros (excluding VAT) for the year 2023.

The same procedural cost of a single-chamber pacemaker was considered for both strategies, with additional costs for the EnSite X patches and technical support for implantation with the EAM. Given that the EAM is currently used for other types of interventions, it was assumed in the base case that the acquisition of the equipment is not necessary.

Regarding fluoroscopy, although the technique based on the EAM aims to minimize its use, the administration of a small dose may be necessary, so this cost is included in both the comparator and the evaluated strategy. The SR showed significant differences in administration time between both technologies, so the cost per minute of fluoroscopy used was estimated. The cost of acquiring the x-ray tube necessary to administer fluoroscopy was annualized to estimate a value distributed over time, by calculating the equivalent annual cost using the following formulas (21):

$$\begin{array}{c} K-\frac{S}{{\left(1+r\right)}^{n}}\\ \begin{array}{c} E=\bar{A(n,r)}\\ A(n,r)=\frac{1-{\left(1+r\right)}^{-n}}{r}\; \end{array} \end{array}$$

Where *S* is the resale value, *n* the years of useful life of the equipment, *r* the interest rate (3%, according to the recommendations of López-Bastida et al. (27), *A(n, r)* is the annuity factor (*n* years at an interest rate *r*), *K* the acquisition price or initial disbursement and *E* the equivalent annual cost (see Supplementary Table S2.1).

The equivalent annual cost was divided by the total time of the interventions in a year to calculate the cost per minute. The average time of implantation with fluoroscopy (obtained from the SR of effectiveness and safety) was used, given that there are no significant differences in total duration between the two types of interventions. The authors used the total time of the intervention because we assumed the x-ray could not be used for other interventions during this whole time.

Finally, the cost per minute was multiplied by the administration time of fluoroscopy in each strategy.

#### 2.2.2 Sensitivity analysis

A one-way deterministic sensitivity analysis was performed, varying the key parameters by ±20% of the mean and adjusting the target population by ±50%. The assumption that fluoroscopy was not used in EAM was also analyzed.

Additionally, a scenario analysis was designed in which an EAM is acquired. In this case, the equivalent annual cost of acquiring the equipment was calculated and divided by the number of patients treated in a year (Supplementary Table S2.2), to determine the equivalent cost per patient.

Furthermore, according to the Abbott Laboratories company, the equipment has an annual maintenance cost of €45,000, which was applied from the second year (after the 1-year warranty provided by the company expired) until the end of the machine's useful life (5 years). In order to calculate the maintenance cost per patient, the annual maintenance costs over the equipment's useful life were summed and divided by 1,035, corresponding to the number of patients treated during this period (=207 patients/year × 5 years of useful life).

## 3 Results

### 3.1 Systematic review

**Figure 1:**
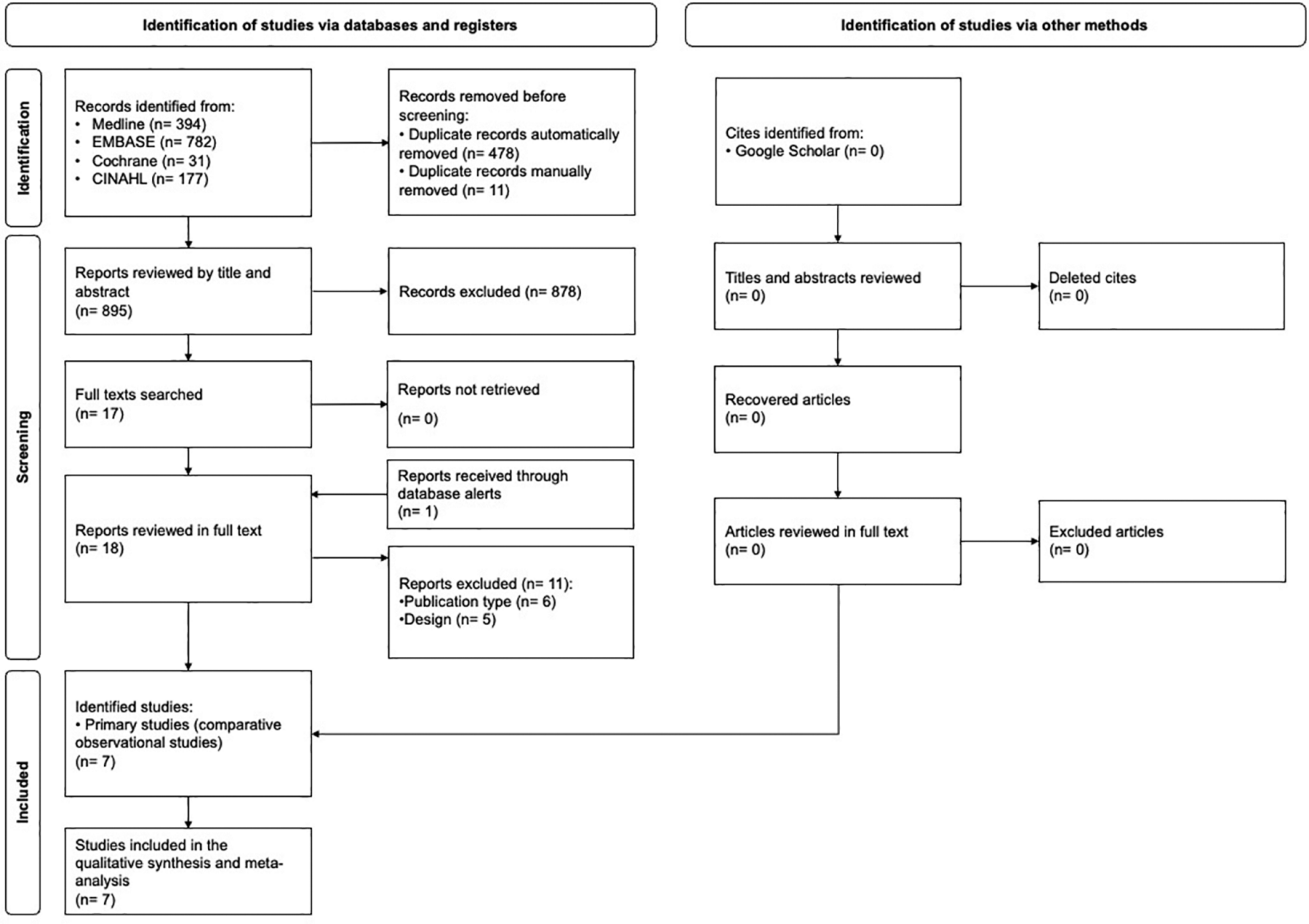
PRISMA flow chart of the study selection process.

The results of the literature search and study selection process are shown in Figure 1. The database search yielded a total of 895 references after deduplication. After screening title and abstract, 18 publications were selected for full-text analysis. According to pre-established selection criteria, 11 of these were excluded. The list of excluded studies at the full-text level, along with the main reason for exclusion, can be found in Supplementary Table S3.

Examination of the bibliographic listing of selected studies and the Google Scholar search did not lead to any additional included studies. No new relevant studies published after the consultation date were found through the alerts (up to October 1, 2023). Therefore, the final selection consisted of seven studies (14, 15, 28–32), all of them on the evaluation of the effectiveness and safety of EAM to guide CSP implantation. No economic evaluation was included in the SR.

#### 3.1.1 Description of included studies

Details of the included studies are summarized in Table 1. All studies were comparative observational studies, most with prospective recruitment and follow-up ranging from 1 to 11 months. These studies were published between 2019 and 2023 and spanned various geographic locations, including China (28, 32), the United States (14, 30), Germany (15), India (29), and Italy (31). The EAMs used were KODEX-EPD (28, 31, 32), ENSITE (15, 30) and CARTO 3 (14); one study did not report the EAM used (29).

**Table 1:** Main characteristics of the included studies on effectiveness and safety.

Author year	N	Women (%)	Age^a^	Pacemaker indication	Comorbidities	Paced QRS duration (ms)^a^	LVEF (%)
Gupta et al. (2022) (29)	EAM: 9 FLU: 8	EAM: NR FLU: NR	EAM: NR FLU: NR	NR	NR	NR	NR
Hua et al. (2021) (28)	EAM: 10 FLU: 10	EAM: 35 FLU: 40	EAM: 55.4 ± 15.3 FLU: 57.6 ± 16.2	SND: 7 (35%) AVB: 13 (65%)	1. Hypertension: 9 (45%) 2. Diabetes mellitus: 4 (20%) 3. Coronary heart disease: 5 (25%)	EAM: 111.2 ± 18.5 FLU: 115.7 ± 20.2	EAM: 59.4 ± 5.5 FLU: 60.5 ± 6.1
Jiménez et al. (2022) (30)	EAM: 10 FLU: 10	EAM: 60 FLU: 60	EAM: 15.5 (8–33) FLU: 13 (8–39)	AVB: 6 (66.7%); SND: 3 (33.3%);	NR	NR	NR
Scarà et al. (2023) (31)	EAM: 22 FLU: 24	EAM: 32 FLU: 42	EAM: 79 ± 10 FLU: 74 ± 8	SND: 13 (28%); AVB: 21 (46%); CRT: 12 (26%)	1. Hypertension: 20 (43%) 2. Ischemic heart disease: 9 (19%) 3. Dilated cardiomyopathy: 12 (26%)	EAM: 102 ± 16 FLU: 110 ± 18	EAM: 54 ± 14 FLU: 51 ± 13
Sharma et al. (2019) (14)	EAM: 29 FLU: 29	EAM: 40 FLU: 50	EAM: 70 ± 14 FLU: 72 ± 11	SND: 15 (50%) AVB: 15 (50%)	1. Hypertension: 29 (97%) 2. Diabetes mellitus: 10 (33%) 3. Coronary heart disease: 12 (40%) 4. Atrial fibrillation: 14 (47%)	EAM: 106 ± 30 FLU: 115 ± 36	EAM: 54 ± 10 FLU: 54 ± 7
Richter et al. (2021) (15)	EAM:29 FLU: 29	EAM: 29 FLU: 28	EAM: 73 ± 13 FLU: 71 ± 13	AVB: 29 (50%) SND: 8 (14%) DCM: 14 (24%)	1. Atrial fibrillation: 27 (47%) 2. Hypertension: 48 (83%) 3. Coronary artery disease: 15 (26%) 4. Ischemic cardiomyopathy: 4 (7%) 5. Non-ischemic cardiomyopathy: 13 (22%) 6. Atrioventricular block: 5 (9%) 7. Mitral valve replacement or repair: 2 (3%).	EAM: 127 ± 34 FLU: 136 ± 39	EAM: 54 ± 13 FLU: 54 ± 13
Wang et al. (2023) (32)	EAM: 20 FLU: 20	EAM: 30 FLU: 35	EAM: 59.6 ± 12.2 FLU: 62.3 ± 18.9	SND: 23 (57.5%) AVB: 27 (67.5%) DCM: 1 (2.5%)	NR	EAM: 131.2 ± 12.5 FLU: 130.1 ± 8.7	EAM: 57.9 ± 9.1 FLU: 58.3 ± 6.9

AVB, atrioventricular block; CTR, cardiac resynchronization therapy; FLU, fluoroscopy group; DCM, dilated cardiomyopathy; EAM, electro-anatomical mapping-guided system group; ms, milliseconds; NR, not reported; SND, sinus node dysfunction.

^a^ Mean ± SD.

Reported outcome measures included successful procedure, procedural details, pacing parameters, left ventricular ejection fraction, and complications. A total of 231 patients were enrolled across the seven studies, with an average sample size of 32 patients, ranging from 17 to 54 participants in the individual studies. Of the total recruits, 80 were women (35%), with a mean age of 58.5 years (SD = 11). Among the recruited patients, 48.9% received pacemakers for AVB and 30.4% for SND.

#### 3.1.2 Risk of bias in included studies

**Figure 2:**
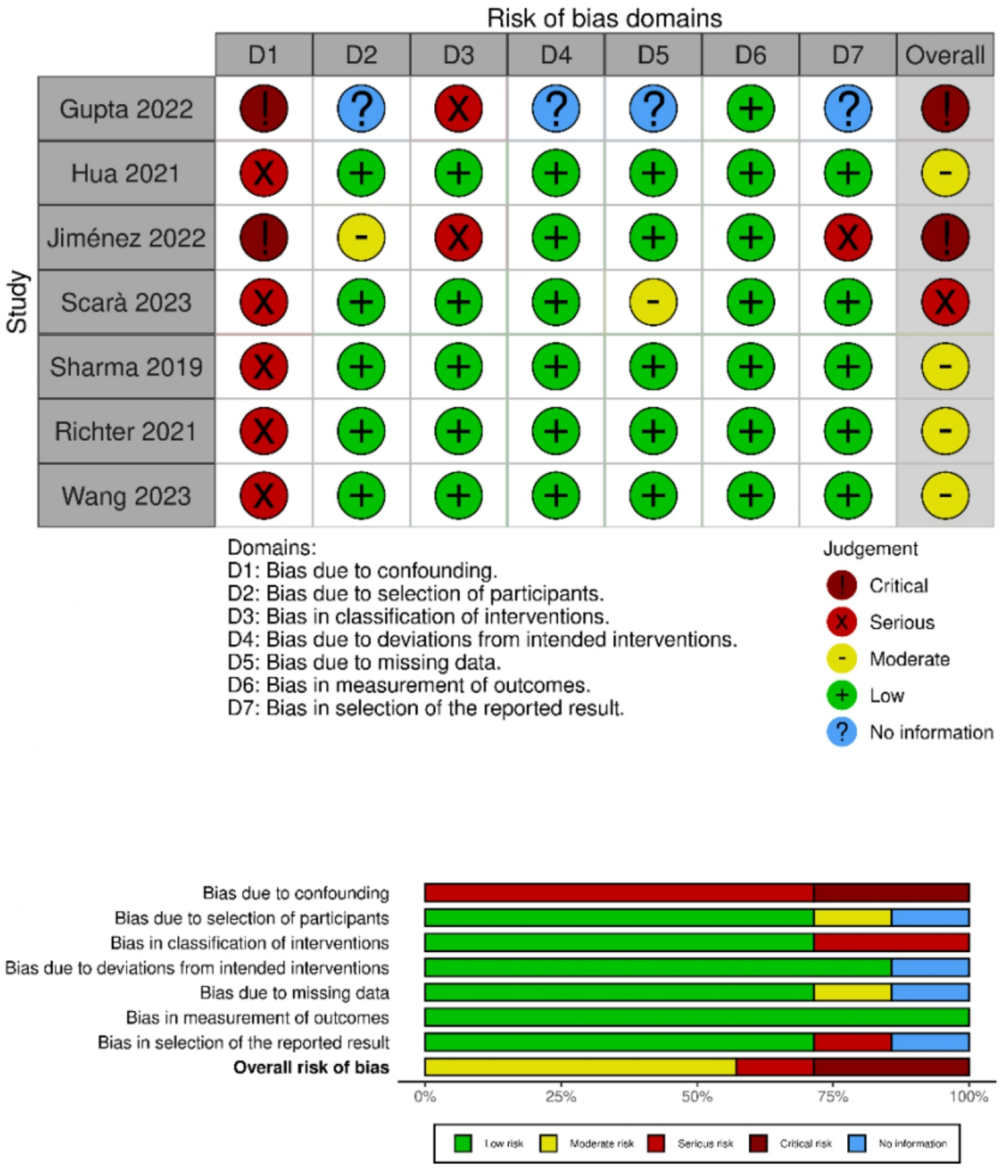
Risk of bias summary for the included studies.

The results of the risk of bias assessment in the included studies are shown in Figure 2. Five of the seven studies showed a serious risk of bias in the domain addressing confounding variables, while the remaining two demonstrated a critical risk of bias. In the other domains, most studies showed a low risk of bias, except for those conducted by Gupta et al. (29), Jiménez et al. (30), and Scarà et al. (31).

Gupta et al. (29) raised concerns about a serious risk of bias in the domain related to the classification of interventions and lacked clear information to evaluate domains 4, 5, and 7 (30) presented a moderate risk of bias in the participant selection domain, and Scarà et al. (31) showed a moderate risk of bias due to the selection of reported results.

#### 3.1.3 Publication bias

A funnel plot analysis and Egger's test could not be performed because the minimum number of studies required to assess the publication bias for any of the outcomes was not reached (*n* = 10).

#### 3.1.4 Summary of results

The results of the meta-analysis conducted are available in the Supplementary Table S4.

#### 3.1.5 Procedure details during implantation

**Figure 3:**
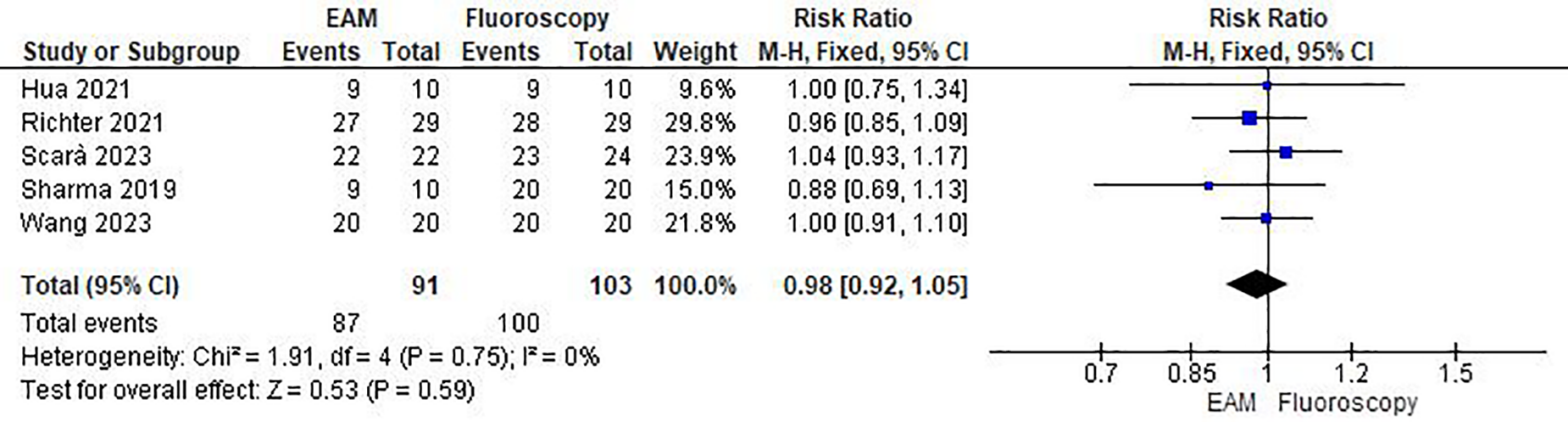
Forest plot of successful procedure.

*Successful procedure.* No statistically significant differences were observed between EAM and fluoroscopy (RR = 0.98; 95% CI: 0.92–1.05; *p* = 0.59; *k* = 5; *I*^2^ = 0%) (see Figure 3).

**Figure 4:**
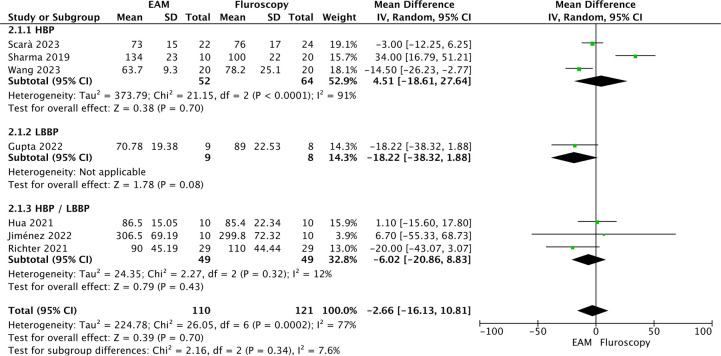
Forest plot of procedural time (minutes).

*Procedural time [minutes (min.)].* No statistically significant differences were observed between EAM and fluoroscopy (MD = −2.66 min.; 95% CI: −16.13–10.81; *p* = 0.70; *k* = 7; *I*^2^ = 77%). In the subgroup analysis, no statistically significant differences were observed between HBP, LBPP and HBP/LBPP subgroups (test for subgroup differences: Chi^2^ = 2.16; df = 2; *p* = 0.34; *I*^2^ = 7.6%) (See Figure 4). In the sensitivity analysis, heterogeneity was reduced to 12% when the study by Sharma et al. (14) was excluded, and the result became statistically significant. A reduction in procedural time was observed in favor of the EAM (MD = −8.37 min.; 95% CI: −15.19 to −1.55; *p* = 0.02; *k* = 6, *I*^2^ = 12%) and no statistically significant differences were observed between HBP, LBPP and HBP/LBPP subgroups (test for subgroup differences: Chi^2^ = 0.98; df = 2; *p* = 0.6; *I*^2^ = 0%).

**Figure 5:**
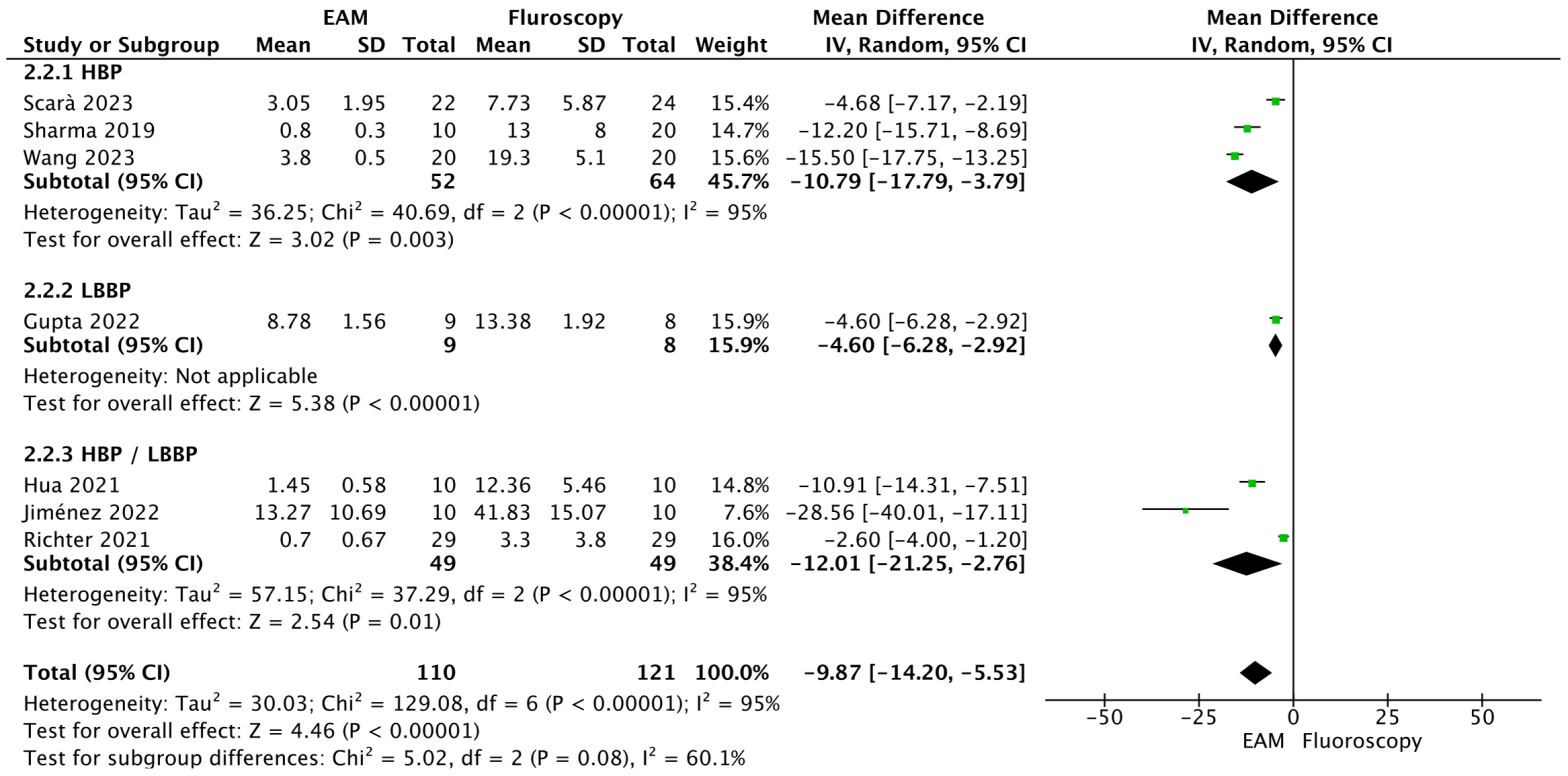
Forest plot of total fluoroscopy time (minutes).

*Total fluoroscopy time (min.).* Statistically significant differences were observed in favor of EAM. Fluoroscopy time was shorter in the EAM group (MD = −9.87 min.; 95% CI: −14.20 to −5.53; *p* < 0.001; *k* = 7; *I*^2^ = 95%). In the subgroup analysis, no statistically significant differences were observed between HBP, LBPP and HBP/LBPP subgroups (test for subgroup differences: Chi^2^ = 5.02; df = 2; *p* = 0.08; *I*^2^ = 60.1%) (see Figure 5). In the sensitivity analysis, none of the studies appeared to be responsible for the heterogeneity; study-by-study exclusion did not reduce heterogeneity.

*His lead fluoroscopy time (min.).* A reduction was observed in favor of the EAM group (MD = −8.08 min.; 95% CI: −10.36 to −5.81; *p* < 0.001; *k* = 2; *I*^2^ = 0%) (see Supplementary Figure S4.1).

*Total fluoroscopy dose (mGy).* No statistically significant differences were observed between the groups (MD = −55.28 mGy; 95% CI: −118.67 to 8.12; *p* = 0.09; *k* = 3; *I*^2^ = 97%) (see Supplementary Figure S4.2). In the sensitivity analysis, heterogeneity was reduced to 18% when the study by (28) was excluded, and the result became statistically significant (MD = −90.23 mGy; 95% CI: −124.90 to −55.55; *p* < 0.001; *k* = 2; *I*^2^ = 18%). Similarly, heterogeneity was reduced to 0% when the study by (32) was excluded (MD = −22.35 mGy; 95% CI: −29.28 to −15.41; *p* < 0.001; *k* = 2; *I*^2^ = 0%).

*His lead fluoroscopy dose (mGy).* A reduction of 17 mGy was observed in favor of the EAM group (MD = −17.21 mGy; 95% CI: −24.08 to −10.34; *k* = 1; *p* < 0.001) (see Supplementary Figure S4.3).

*Paced QRS duration [time in milliseconds (ms) from the beginning of the Q or R wave to the end of the R or S wave].* No significant differences were observed between EAM and fluoroscopy (MD = −3.92 ms; 95% CI: −9.43 to 1.60; *p* = 0.16; *k* = 4; *I*^2^ = 0%) (see Supplementary Figure S4.4).

*His-ventricular interval duration (period between the onset of the His bundle spike and the earliest ventricular activation) (ms).* No significant differences were observed between EAM and fluoroscopy (MD = −1.04 ms; 95% CI: −7.19 to 5.12; *p* = 0.74; *k* = 2; *I*^2^ = 0%) (see Supplementary Figure S4.5).

#### 3.1.6 Stimulation parameters during implantation

*Capture threshold [the minimum amount of energy from an electrical impulse that is required to produce myocardial contraction (V/1 ms) during implantation].* No statistically significant differences were observed between EAM and fluoroscopy (MD = −0.02 V/1 ms; 95% CI: −0.15 to 0.12; *p* = 0.82; *k* = 6; *I*^2^ = 63%) (see Supplementary Figure S4.6).

*Impedance [assessing the integrity of pacemaker pacing and sensing leads (Ω)].* No statistically significant differences were observed between EAM and fluoroscopy (MD = 16.51 *Ω*; 95% CI: −15.28 to 48.30; *p* = 0.31; *k* = 5; *I*^2^ = 16%) (see Supplementary Figure S4.7).

*R-wave amplitude [the amplitude of the intracardiac signal detected by the device (mV)].* No statistically significant differences were observed between EAM and fluoroscopy (MD = 0.46 mV; 95% CI: −0.15 to 1.07; *p* = 0.14; *k* = 6; *I*^2^ = 0%) (see Supplementary Figure S4.8).

#### 3.1.7 Stimulation parameters during follow-up

*Capture threshold (follow-up ranged from 1 to 6 months).* No statistically significant differences were observed between EAM and fluoroscopy (MD = −0.08 V/1 ms; 95% CI: −0.23 to 0.07; *p* = 0.32; *k* = 4; *I*^2^ = 24%) (see Supplementary Figure S4.9).

*Impedance (follow-up ranged from 3 to 6 months).* No statistically significant differences were observed between EAM and fluoroscopy (MD = −7.31 *Ω*; 95% CI: −50.17 to 35.55; *p* = 0.74; *k* = 2; *I*^2^ = 0%) (see Supplementary Figure S4.10).

*R-wave amplitude (follow-up ranged from 1 to 6 months).* No statistically significant differences were observed between EAM and fluoroscopy (MD = −0.26 mV; 95% CI: −0.16 to 0.65; *p* = 0.58; *k* = 6; *I*^2^ = 0%) (see Supplementary Figure S4.11).

*Left ventricular ejection fraction (LVEF) (%) at follow-up (3–6 months).* No statistically significant differences were observed between EAM and fluoroscopy (MD = 0.44%; 95% CI: −3.85 to 4.74; *p* = 0.84; *k* = 2; *I*^2^ = 57%) (see Supplementary Figure S4.12).

#### 3.1.8 Complications

Five of the included studies reported immediate procedure-related complications, with no complications observed in the patients (see Table 2). Regarding other complications recorded during follow-up (ranging from 1 to 6 months), only one case of lead displacement and one case of increased capture threshold were observed in the fluoroscopy group.

**Table 2:** Reported complications in included studies.

Studies	Complication	Electro-anatomical mapping-guided system		Fluoroscopy		Follow-up
		No. events	No. total	No. events	No. total	
Hua et al. (2021) (28), Jiménez et al. (2022) (30), Richter et al. (2021) (15), Scarà et al. (2023) (31), Wang et al. (2023) (32)	Procedure-related complications	0	91	0	93	Implantation
Richter et al. (2021) (15), Scarà et al. (2023) (31), Sharma et al. (2019) (14), Wang et al. (2023) (32)	Lead displacement	0	81	1	93	1–6 months
Scarà et al. (2023) (31)	Lead revision	0	22	0	24	6 months
Sharma et al. (2019) (14)	Pneumothorax	0	10	0	20	1 month
	Pericardial effusion	0	10	0	20	1 month
	Increase in capture threshold	0	10	1	20	1 month
	Device infection	0	10	0	20	1 month

N, number.

#### 3.1.9 Certainty of evidence

The certainty of evidence for all key outcomes was considered very low due to the serious risk of bias in the included studies (see evidence profile in Supplementary Table S5).

### 3.2 Economic evaluation

#### 3.2.1 Parameter values

Parameters used in the cost analysis can be consulted in Table 3.

**Table 3:** Parameters used in the cost analysis.

Parameters	
Cost of the procedure, implantation using fluoroscopy (€, without VAT)	2,847.98
Cost of the procedure, implantation via EAM (€, without VAT)	4,247.98
Equivalent cost per patient of the Ensite EAM (€, without VAT)	179.33
Maintenance cost per patient of the Ensite EAM (€, without VAT)	161.61
Equivalent cost per minute of the x-ray tube for the administration of fluoroscopy (€, without VAT)	0.23
Fluoroscopy time, fluoroscopy implantation (minutes)	15.84
Fluoroscopy time, implantation via EAM (minutes)	4.55

EAM, electro-anatomical mapping-guided system.

#### 3.2.2 Base case

The results of the analysis show that the implantation of CSP using an Ensite X EAM would entail a cost of €4,248.86 per patient, while the cost of using the fluoroscopy technique is €2,851.06, resulting in an increase of €1,397.81 per patient (see Table 4).

**Table 4:** Results of the cost analysis (base case). Cost per patient.

Concept	Fluoroscopy	Electro-anatomical mapping-guided system	Incremental cost
Procedure (€)	2,847.98	4,247.98	1,400
Fluoroscopy administration (€)	3.08	0.88	−2.19
Costs per patient (€)	2,851.06	4,248.86	1,397.81

#### 3.2.3 Sensitivity analysis

The sensitivity analysis shows that the incremental cost per patient can vary between €548.21 and €2,247.40 (Table 5). This variation is mainly due to the differences in the cost of the procedure with the EAM technique, specifically the costs associated with Ensite patches and technical support, which are the differential resources between the two compared techniques.

**Table 5:** Results of the cost analysis (sensitivity analysis). Cost per patient.

Parameter	Value in the base case	New value	Incremental cost
Cost of the procedure, implantation via EAM(€)	4,247.98	3,398.38 [Assumption, −20%]	548.21
		5,097.58 [Assumption, +20%]	2,247.40
Cost of the x-ray tube for the fluoroscopy administration (€)	30,000	24,000 [Assumption, −20%]	1,398.25
		36,000 [Assumption, +20%]	1,397.37
Fluoroscopy time, fluoroscopy implantation (minutes)	15.84	12.67 [Assumption, −20%]	1,398.42
		19.01 [Assumption, +20%]	1,397.19
Fluoroscopy time, implantation via EAM (minutes)	4.55	0 [Assumption]	1,396.92
		3.64 [Assumption, −20%]	1,397.63
		5.46 [Assumption, +20%]	1,397.98
Target population	207	103.5 [Assumption, −50%]	1,395.61
		310.5 [Assumption, +50%]	1,398.54

EAM, electro-anatomical mapping-guided system.

#### 3.2.4 Scenario analysis

Finally, if a hospital needed to acquire the necessary equipment for EAM, the incremental cost would increase to €1,738.74 per patient (Table 6).

**Table 6:** Results of the cost analysis (scenario analysis). Cost per patient.

Concept	Fluoroscopy	Electro-anatomical mapping-guided system	Incremental cost
Procedure (€)	2,847.98	4,247.98	1,400
Fluoroscopy administration (€)	3.08	0.88	−2.19
Ensite EAM (€)	0	179.33	179.33
Maintenance of the EAM (€)	0	161.61	161.61
Cost per patient (€)	2,851.06	4,589.80	1,738.74

EAM, electro-anatomical mapping-guided system.

## 4 Discussion

This SR on effectiveness and safety identified seven observational studies with control groups (*N* = 231), published between 2019 and 2023, comparing EAM with fluoroscopy for CSP in patients with bradyarrhythmia requiring permanent pacemakers (symptomatic SND or AVB). The included studies predominantly evaluated HBP, with only two studies (29, 30) reporting LBBP or a mix of HBP and LBBP. While both modalities fall under the broader category of CSP and aim to replicate physiological ventricular activation, the procedural differences between HBP and LBBP may influence outcomes such as procedural time and fluoroscopy use. On the other hand, while our target population was patients with symptomatic bradyarrhythmias requiring pacemakers (SND or AVB), some included studies also incorporated patients with indications for CRT, such as in (15) and Scarà et al. (31). To maintain the relevance of our review, we included studies with mixed populations only if the results for the SND or AVB subgroup were reported separately, or if these patients represented approximately 80% of the study population. This criterion ensured the applicability of our findings while acknowledging the growing use of CSP for CRT indications, particularly in cases of left bundle branch block (LBBB), where the complexity of lead implantation may influence outcomes. In CRT cases, particularly those with LBBB, the success of QRS narrowing depends largely on the location of the conduction block rather than the guidance method (EAM vs. fluoroscopy) (33). Furthermore, lead implantation in dilated hearts presents additional technical challenges, which could influence procedural success rates and fluoroscopy times. Future studies focusing on these subpopulations are needed to better understand the role of CSP and EAM guidance in complex CRT cases.

Although the use of CSP and EAM is increasing, well-conducted RCTs evaluating their use vs. fluoroscopy are not available.

The authors did not find studies that evaluated the use of EAM vs. fluoroscopy for CSP implantation in populations truly vulnerable to radiation, such as pregnant women or pediatric patients (5), with the exception of the study by Jimenez et al. (30), which included some pediatric patients within its cohort. This gap is particularly important, as the use of EAM, which significantly reduces fluoroscopy time, has the potential to offer substantial safety benefits for vulnerable populations. Pediatric patients and pregnant women, for example, face heightened risks from radiation exposure due to their increased susceptibility to long-term adverse effects, including carcinogenesis and teratogenesis (34–37). For such groups, EAM could become a preferred approach, emphasizing the need for targeted research to validate its safety and efficacy in these populations.

Our meta-analysis revealed that EAM has similar effectiveness and safety to fluoroscopy in guiding the implantation of CSP for the treatment of bradyarrhythmia. No statistically significant differences were observed in terms of successful procedure, procedural details, or pacing parameters between EAM and fluoroscopy. One significant finding was a shorter total fluoroscopy time with EAM, with a reduction of 9.87 min. This finding is in line with indications for reducing fluoroscopy exposure (13–15). However, no significant differences were observed in the total fluoroscopy dose, possibly due to the small number of studies (three) analyzing this outcome, improvements in x-ray generator software using low-frequency fluoroscopy, new generators, and technological advances in the leads (38). Another significant finding was a shorter His lead fluoroscopy time with EAM, with a reduction of 8.08 min, and a lower His lead fluoroscopy dose with EAM, with a reduction of 17.21 mGy. Regarding safety, no differences were observed in procedure-related complications or other complications such as lead displacement, pneumothorax, device infection, or pericardial effusion. In addition to the benefits observed with EAM, the integration of visualizable sheaths specifically designed for cardiac electrophysiology procedures offers another potential avenue to reduce fluoroscopy exposure further. Recent studies (39, 40) have shown that these sheaths enable more precise navigation without reliance on fluoroscopy, making them a promising enhancement for EAM-guided implantations. Incorporating such devices in future studies could refine the overall strategy, enhancing safety while minimizing radiation risks.

The high heterogeneity observed in fluoroscopy time (*I*^2^ = 95%) likely reflects variability across studies due to a combination of factors, including differences in operator experience, delivery system selection, and procedural complexity. For instance, fluoroscopy times reported in Jimenez et al. (41.83 ± 15.07 min) were notably higher compared to Sharma et al. (13 ± 8 min), underscoring the influence of differing protocols and operator expertise. Procedural times for HBP are consistently longer than for LBBP due to the technical complexity of His bundle localization (41). In our review, three studies (14, 31, 32) exclusively performed HBP, whereas Gupta et al. (29) focused solely on LBBP, which may explain some of the observed heterogeneity. As noted in the Results section, we performed a subgroup analysis stratifying studies by pacing modality (HBP, LBBP, or mixed HBP/LBBP). This analysis revealed no statistically significant differences in fluoroscopy time between these subgroups, suggesting that pacing modality alone does not account for the observed variability. Additionally, we performed a sensitivity analysis by systematically excluding each study to assess its impact on the heterogeneity. This analysis did not identify any single study as a major contributor, indicating that the heterogeneity persists due to a combination of factors. Future research should consistently report factors such as operator-specific experience and procedural protocols to enable more precise analyses and reduce unexplained variability. Therefore, the available evidence indicates that CSP pacemaker implantation using EAM has similar effectiveness and safety to implantation with fluoroscopy and reduces fluoroscopy time. However, no significant differences were observed in the total fluoroscopy dose.

No economic evaluations comparing EAM with fluoroscopy were identified in this SR. The cost analysis, conducted from the perspective of the National Health Service, shows that replacing fluoroscopy with Ensite X EAM would increase the cost of the CSP implantation procedure by €1,397.81 per patient compared to current clinical practice of implantation with fluoroscopy. This cost could rise to €1,738.74 per patient if a hospital had to assume the investment in EAM equipment and its annual maintenance.

The strength of the SR of the literature on safety, effectiveness, and cost-effectiveness is related to the fact that it was conducted in accordance with the fundamental principles of SRs to ensure transparency, replicability and ease of updating. The explicit information on the methodology used and the availability of the extracted data mean that it can also be used as the object of a critical evaluation. Furthermore, to the best of the authors' knowledge, it is the only SR carried out to date on the effect of using EAM vs. fluoroscopy to guide CSP implantation in the treatment of bradyarrhythmias with indication for a permanent pacemaker (symptomatic SND or BAV).

One of the main limitations of this SR is the possibility that relevant studies were not included in the analysis because of their not having been published, because they were published in a language other than English or Spanish or because they have been published in unindexed journals.

Other limitations are related to the characteristics of the included studies. One limitation is the different intracardiac navigators used in the included studies, which may have important differences between them. On the other hand, only observational studies with a control group could be identified and some studies involved mixed populations (SND, AVB, and CRT indications). However, we applied strict inclusion criteria, ensuring that results were either reported separately for our target population or that this subgroup comprised at least 80% of the study participants. Nevertheless, the complexity of lead implantation in CRT cases, particularly for patients with dilated cardiomyopathy or left bundle branch block, may have influenced procedural success and fluoroscopy times. Another limitation is the substantial heterogeneity observed in fluoroscopy time, which persisted despite sensitivity and subgroup analyses. The lack of significant differences in fluoroscopy time between pacing modalities (HBP, LBBP, or mixed HBP/LBBP) suggests that the variability likely arises from unreported factors such as operator experience, delivery system selection, and procedural complexity. Consistent reporting of these factors in future research will be crucial to reducing unexplained variability and improving the interpretability of meta-analyses.

Another limitation is that the studies included in this review primarily involved patients with standard anatomy, with only a small subset reporting cases with DCM or other complex anatomical features. As the EHRA highlights the potential advantages of EAM in complex cases, such as congenital heart disease or post-failed BiV implants, further research is needed to evaluate the clinical and economic outcomes of EAM in these populations.

Finally, the small number of included studies, affecting the ability to assess publication bias. We emphasize the need for additional studies in the future to expand the available evidence base and allow a more robust assessment of publication bias in this area of research. Furthermore, several studies included variables that are not relevant for health decision-making. An important consideration for future research is to conduct well-designed observational studies and RCTs in homogeneous patient populations to enhance the quality of evidence and provide more appropriate responses.

In addition to the SR, a partial economic evaluation was developed based on a cost analysis for Spain. A complete economic evaluation, comparing costs and effects, could not be carried out because the evaluated technology (EAM for the implantation of CSP pacemakers) did not demonstrate any health effect, although it was concluded that the total fluoroscopy time is shorter. The costs included in the analysis are limited to those at the time of implantation.

Furthermore, only the device manufactured by Abbott Laboratories was analyzed due to the lack of cost information for devices from other companies. The cost of the fluoroscopy tube was based on an approximate price reported by expert contributors to this study. Regarding the estimation of the target population, real data on the vulnerable population receiving CSP pacemakers were unavailable, so a 2% value reported by the industry was used. However, these data were modified in the sensitivity analysis to assess their impact on the incremental cost per patient.

In addition, cross-validation could not be performed as the results of this analysis could not be compared with those of other studies, due to the absence of previous economic evaluations that met the SR inclusion criteria. However, the assumptions made were validated by experts (face validation).

## 5 Conclusion

This SR provides the first comprehensive comparison of EAM and fluoroscopy for CSP lead implantation in bradyarrhythmia patients requiring permanent pacemakers. Evidence from seven observational studies indicates similar effectiveness and safety for both approaches, with EAM showing significant reductions in overall fluoroscopy time and time and dose for His lead placement. However, the included studies primarily examined patients with standard anatomy, limiting the generalizability of these findings to populations with more complex anatomical conditions. The economic analysis revealed higher costs associated with EAM, highlighting the need for further research to validate its clinical and economic viability, particularly in patients with complex anatomy.

## Data Availability

The original contributions presented in the study are included in the article/Supplementary Material, further inquiries can be directed to the corresponding author.

## References

[1] Kossmann CE. The normal electrocardiogram. Circulation. (1953) 8(6):920–36. 10.1161/01.CIR.8.6.920 13106913

[2] Honarbakhsh S Hunter L Chow A Hunter RJ. Bradyarrhythmias and pacemakers. Br Med J. (2018) 360:k642. 10.1136/bmj.k642 29545242

[3] Barold SS. Cardiac pacing in special and complex situations. Indications and modes of stimulation. Cardiol Clin. (1992) 10(4):573–91. 10.1016/S0733-8651(18)30203-0 1423374

[4] Epstein AE DiMarco JP Ellenbogen KA Estes NAM Freedman RA Gettes LS 2012 ACCF/AHA/HRS focused update incorporated into the ACCF/AHA/HRS 2008 guidelines for device-based therapy of cardiac rhythm abnormalities: a report of the American College of Cardiology foundation/American Heart Association task force on practice guidelines and the heart rhythm society. J Am Coll Cardiol. (2013) 61(3):e6–75. 10.1016/j.jacc.2012.11.007 23265327

[5] Burri H Jastrzebski M Cano Ó Čurila K de Pooter J Huang W EHRA clinical consensus statement on conduction system pacing implantation: endorsed by the Asia Pacific Heart Rhythm Society (APHRS), Canadian Heart Rhythm Society (CHRS), and Latin American Heart Rhythm Society (LAHRS). Europace. (2023) 25(4):1208–36. 10.1093/europace/euad043 37061848 PMC10105878

[6] Vijayaraman P Dandamudi G Zanon F Sharma PS Tung R Huang W Permanent his bundle pacing: recommendations from a multicenter his bundle pacing collaborative working group for standardization of definitions, implant measurements, and follow-up. Heart Rhythm. (2018) 15(3):460–8. 10.1016/j.hrthm.2017.10.039 29107697

[7] Glikson M Nielsen JC Kronborg MB Michowitz Y Auricchio A Barbash IM 2021 ESC guidelines on cardiac pacing and cardiac resynchronization therapy. Eur Heart J. (2021) 42(35):3427–520. 10.1093/eurheartj/ehab364 34455430

[8] Glikson M Nielsen JC Kronborg MB Michowitz Y Auricchio A Barbash IM Guía ESC 2021 sobre estimulación cardiaca y terapia de resincronización. Rev Esp Cardiol. (2022) 75(5):430.e1–86. 10.1016/j.recesp.2021.10.025 35525571

[9] Chen J Einstein AJ Fazel R Krumholz HM Wang Y Ross JS Cumulative exposure to ionizing radiation from diagnostic and therapeutic cardiac imaging procedures: a population-based analysis. J Am Coll Cardiol. (2010) 56(9):702–11. 10.1016/j.jacc.2010.05.014 20619569 PMC2952402

[10] Miller DL Balter S Schueler BA Wagner LK Strauss KJ Vañó E. Clinical radiation management for fluoroscopically guided interventional procedures. Radiology. (2010) 257(2):321–32. 10.1148/radiol.10091269 20959547

[11] Houmsse M Daoud EG. Radiation exposure: a silent complication of catheter ablation procedures. Heart Rhythm. (2012) 9(5):715–6. 10.1016/j.hrthm.2012.01.015 22289168

[12] Wong YM Koh CWY Lew KS Chua CGA Nei W Tan HQ A review on fetal dose in radiotherapy: a historical to contemporary perspective. Phys Med. (2023) 105:102513. 10.1016/j.ejmp.2022.102513 36565555

[13] Lemery R. Interventional electrophysiology at the crossroads: cardiac mapping, ablation and pacing without fluoroscopy—LEMERY—2012. J Cardiovasc Electrophysiol. (2012) 23(10):1087–91. 10.1111/j.1540-8167.2012.02373.x 22882722

[14] Sharma PS Huang HD Trohman RG Naperkowski A Ellenbogen KA Vijayaraman P. Low fluoroscopy permanent his bundle pacing using electroanatomic mapping: a feasibility study. Circ Arrhythm Electrophysiol. (2019) 12(2):e006967. 10.1161/CIRCEP.118.006967 30704289

[15] Richter S Ebert M Bertagnolli L Gebauer R Lucas J Scheller D Impact of electroanatomical mapping-guided lead implantation on procedural outcome of his bundle pacing. EP Europace. (2021) 23(3):409–20. 10.1093/europace/euaa292 33253376

[16] Higgins J Green S Higgins JPT. Cochrane handbook for systematic reviews of interventions version 5.1.0. In: Higgins J Green S, editors. London: The Cochrane Collaboration (2011). (Updated March 2011).

[17] Moher D Liberati A Tetzlaff J Altman DG Altman D Antes G Preferred reporting items for systematic reviews and meta-analyses: the PRISMA statement. PLoS Med. (2009) 6(7). 10.1371/journal.pmed.1000097 PMC2707599 19621072

[18] Higgins JPT Altman DG Gøtzsche PC Jüni P Moher D Oxman AD The Cochrane Collaboration’s tool for assessing risk of bias in randomised trials. BMJ. (2011) 343:d5928. 10.1136/bmj.d5928 22008217 PMC3196245

[19] Sterne JA Hernán MA Reeves BC Savović J Berkman ND Viswanathan M ROBINS-I: a tool for assessing risk of bias in non-randomised studies of interventions. BMJ. (2016) 355:i4919. 10.1136/bmj.i4919 27733354 PMC5062054

[20] Higgins JPT Morgan RL Rooney AA Taylor KW Thayer KA Silva RA A tool to assess risk of bias in non-randomized follow-up studies of exposure effects (ROBINS-E). Environ Int. (2024) 186:108602. 10.1016/j.envint.2024.108602 38555664 PMC11098530

[21] Drummond MF Sculpher MJ Torrance GW O’Brien BJ Stoddart GL. Methods for the Economic Evaluation of Health Care Programme. 3rd ed. Oxford: Oxford University Press (2005).

[22] López-de Argumedo M Reviriego E Gutiérrez A Bayón J. Actualización del Sistema de Trabajo Compartido para Revisiones Sistemáticas de la Evidencia Científica y Lectura Crítica (Plataforma FLC 3.0). Informes de Evaluación de Tecnologías Sanitarias. (2017):144–144.

[23] Egger M Smith G Altman DG. Systematic Reviews in Health Care: Meta-Analysis in Context, 2nd Edition |Wiley. https://www.wiley.com/en-us/Systematic+Reviews+in+Health+Care%3A+Meta+Analysis+in+Context%2C+2nd+Edition-p-9780727914880 (Accessed January 26, 2023).

[24] Atkins D Best D Briss PA Eccles M Falck-Ytter Y Flot-torp S Grading quality of evidence and strength of recommendations. BMJ. (2004) 328:1490. 10.1136/bmj.328.7454.1490 15205295 PMC428525

[25] Pombo Jiménez M Chimeno García J Bertomeu González V Cano Pérez Ó. Registro español de marcapasos. XIX informe oficial de la Asociación del Ritmo Cardiaco de la Sociedad Española de Cardiología (2021). Rev Esp Cardiol. (2022) 75(11):949–59. 10.1016/j.recesp.2022.08.006

[26] Husereau D Drummond M Petrou S Carswell C Moher D Greenberg D Consolidated health economic evaluation reporting standards (CHEERS) statement. BMJ. (2013) 346:f1049. 10.1136/bmj.f1049 23529982

[27] López Bastida J Oliva J Antoñanzas F García-Altés A Gisbert R Mar J Propuesta de guía para la evaluación económica aplicada a las tecnologías sanitarias. Gaceta Sanitaria. (2010) 24(2):154–70. 10.1016/j.gaceta.2009.07.011 19959258

[28] Hua W Liu X Gu M Niu HX Chen X Tang M Novel wide-band dielectric imaging system guided lead deployment for his bundle pacing: a feasibility study. Front Cardiovasc Med. (2021) 8:712051. 10.3389/fcvm.2021.712051 34540916 PMC8446512

[29] Gupta A Kataria V Kitey P Nair M. Mapping of left bundle anatomy in left ventricle facilitates left bundle pacing. JACC Clin Electrophysiol. (2022) 8(6):824–7. 10.1016/j.jacep.2022.03.005 35738863

[30] Jimenez E Gordon A Cortez D. Reduction of fluoroscopy in conduction system pacing guided by electroanatomical mapping in pediatrics and congenital heart disease. Indian Pacing Electrophysiol J. (2022) 22(4):182–5. 10.1016/j.ipej.2022.04.005 35447346 PMC9263654

[31] Scarà A Golia P Grieco D Borrelli A De Ruvo E Bressi E Low fluoroscopy permanent his bundle pacing using a new electroanatomic mapping system (KODEX EPD). A multicenter experience. J Arrhythm. (2023) 39(1):18–26. 10.1002/joa3.12803 36733331 PMC9885313

[32] Wang L Yang S Tang B Wang F Sang W Han Y Feasibility, safety and effectiveness of mapping system assisted conduction system pacing: a single-center prospective study. Sci Rep. (2023) 13(1):9683. 10.1038/s41598-023-36546-x 37322082 PMC10272113

[33] Upadhyay GA Cherian T Shatz DY Beaser AD Aziz Z Ozcan C Intracardiac delineation of septal conduction in left bundle-branch block patterns. Circulation. (2019) 139(16):1876–88. 10.1161/CIRCULATIONAHA.118.038648 30704273

[34] Brenner DJ Hall EJ. Computed tomography–an increasing source of radiation exposure. N Engl J Med. (2007) 357(22):2277–84. 10.1056/NEJMra072149 18046031

[35] Brent RL. Saving lives and changing family histories: appropriate counseling of pregnant women and men and women of reproductive age, concerning the risk of diagnostic radiation exposures during and before pregnancy. Am J Obstet Gynecol. (2009) 200(1):4–24. 10.1016/j.ajog.2008.06.032 19121655

[36] Khong PL, ICRP, Ringertz H Donoghue V Frush D Rehani M ICRP publication 121: radiological protection in paediatric diagnostic and interventional radiology. Ann ICRP. (2013) 42(2):1–63. 10.1016/j.icrp.2012.10.001 23218172

[37] Stabin MG. Radiation dose and risks to fetus from nuclear medicine procedures. Phys Med. (2017) 43:190–8. 10.1016/j.ejmp.2017.04.001 28454782

[38] Bru P Dompnier A Amara W Haddad G Galuscan G Sagnol P Radiation exposure during cardiac device implantation: lessons learned from a multicenter registry. Pacing Clin Electrophysiol. (2020) 43(1):87–92. 10.1111/pace.13842 31710385

[39] Janosi K Debreceni D Janosa B Bocz B Simor T Kupo P. Visualizable vs. standard, non-visualizable steerable sheath for pulmonary vein isolation procedures: randomized, single-centre trial. Front Cardiovasc Med. (2022) 9:1033755. 10.3389/fcvm.2022.1033755 36465461 PMC9709402

[40] Khalaph M Sommer P Lucas P Guckel D Fink T Sciacca V First clinical experience using a visualized sheath for atrial fibrillation ablation. Pacing Clin Electrophysiol. (2022) 45(8):922–9. 10.1111/pace.14555 35716400

[41] Hua W Fan X Li X Niu H Gu M Ning X Comparison of left bundle branch and his bundle pacing in bradycardia patients. JACC Clin Electrophysiol. (2020) 6(10):1291–9. 10.1016/j.jacep.2020.05.008 33092757

