# Supplementary material for: Conduction system pacing using electro-anatomical mapping-guided system vs. fluoroscopy: a systematic review, meta-analysis and economic evaluation
Source: Front Cardiovasc Med. 2025 Jan 14;11:1519127. doi: 10.3389/fcvm.2024.1519127 (PMC11796619; doi:10.3389/fcvm.2024.1519127)
Supplement: Supplementary file 1 [file Supplementaryfile1.docx]

Tables S1. Search strategies

| MEDLINE(R) ALL (Ovid) | |
| --- | --- |
| 1 | exp Bundle of His/ |
| 2 | ((His-bundle or His or Branch or conduction system or antybradycardia) adj3 (pacing or bundle*)).ti,ab,kf. |
| 3 | (His bundle pacing or HBP or Left bundle branch pacing or LBBP or Left bundle branch area pacing or LBBAP).ti,ab,kf. |
| 4 | 1 or 2 or 3 |
| 5 | device implantation.kf. |
| 6 | (Electromapping or Electro-mapping or EAM or EAM-guided or 3D-EAM).ti,ab,kf. |
| 7 | ((Intracardiac or intra-cardiac or nonfluoroscop*' or 'without fluoroscop*' or “zero-fluoroscop*” or “near-zero fluoroscop*” or “zero fluoroscop*” or “zero x-ray” or “alternative fluoroscop*” or “fluoroscopy elimination” or “radiation-free” or “fluoroless” or “fluoroscop*-free or non-fluoroscop*) adj5 (navigat* or intervention or electroanatom* or electro-anatom* or electrogram*)).ti,ab,kf. |
| 8 | ((electroanatom* or electro-anatom* or electrogram* or system* or navigat* or tecnique*) adj3 (mapping* or mapping-guide* or guide* or Three-dimension* or Three dimension* or 3D or 3-D)).ti,ab,kf. |
| 9 | (EnSite* or Carto* or NavX* or RPM* or LocaLisa* or Affera* or IntellaNav* or Rhythmia HDx* or KODEX-EPD).ti,ab,kf. |
| 10 | 5 or 6 or 7 or 8 or 9 |
| 11 | 4 and 10 |

| Embase (Elsevier) | |
| --- | --- |
| #1 | 'his bundle'/exp |
| #2 | (('his bundle' OR his OR branch OR 'conduction system' OR antybradycardia) NEAR/3 (pacing OR bundle*)):ti,ab,kw |
| #3 | 'his bundle pacing':ti,ab,kw OR hbp:ti,ab,kw OR 'left bundle branch pacing':ti,ab,kw OR lbbp:ti,ab,kw OR 'left bundle branch area pacing':ti,ab,kw OR lbbap:ti,ab,kw |
| #4 | #1 OR #2 OR #3 |
| #5 | 'device implantation':kw |
| #6 | electromapping:ti,ab,kw OR 'electro mapping':ti,ab,kw OR eam:ti,ab,kw OR 'eam guided':ti,ab,kw OR '3d eam':ti,ab,kw |
| #7 | ((intracardiac OR 'intra cardiac' OR nonfluoroscop* OR 'without fluoroscop*' OR 'zero-fluoroscop*' OR 'near-zero fluoroscop*' OR 'zero fluoroscop*' OR 'zero x-ray' OR 'alternative fluoroscop*' OR 'fluoroscopy elimination' OR 'radiation-free' OR 'fluoroless' OR 'fluoroscop*-free' OR 'non-fluoroscop*') NEAR/5 (navigat* OR intervention OR electroanatom* OR 'electro anatom*' OR electrogram*)):ti,ab,kw |
| #8 | ((electroanatom* OR 'electro anatom*' OR electrogram* OR system* OR navigat* OR tecnique*) NEAR/3 (mapping* OR 'mapping guide*' OR guide* OR 'three dimension*' OR 'three dimension*' OR 3d OR '3 d')):ti,ab,kw |
| #9 | ensite*:ti,ab,kw OR 'carto*':ti,ab,kw OR navx*:ti,ab,kw OR rpm*:ti,ab,kw OR localisa*:ti,ab,kw OR affera*:ti,ab,kw OR intellanav*:ti,ab,kw OR 'rhythmia hdx*':ti,ab,kw OR 'kodex epd':ti,ab,kw |
| #10 | #5 OR #6 OR #7 OR #8 OR #9 |
| #11 | #4 AND #10 |

| CENTRAL (Cochrane Library - Wiley) | |
| --- | --- |
| #1 | MeSH descriptor: [Bundle of His] explode all trees |
| #2 | ((His-bundle or His or Branch or "conduction system" or antybradycardia) NEAR/3 (pacing or bundle*)):ti,ab,kw |
| #3 | (("His bundle pacing" or HBP or "Left bundle branch pacing" or LBBP or "Left bundle branch area pacing" or LBBAP):ti,ab,kw |
| #4 | #1 OR #2 OR #3 |
| #5 | device implantation:kw |
| #6 | (Electromapping or Electro-mapping or EAM or EAM-guided or 3D-EAM):ti,ab,kw |
| #7 | ((Intracardiac or intra-cardiac or nonfluoroscop* or "without fluoroscop*" or "zero-fluoroscop*" or "near-zero fluoroscop*" or "zero fluoroscop*" or "zero x-ray" or "alternative fluoroscop*" or "fluoroscopy elimination" or "radiation-free" or "fluoroless" or "fluoroscop*-free" or non-fluoroscop*) NEAR/5 (navigat* or intervention or electroanatom* or electro-anatom* or electrogram*)):ti,ab,kw |
| #8 | ((electroanatom* or "electro-anatom*" or electrogram* or system* or navigat* or tecnique*) NEAR/3 (mapping* or "mapping-guide*" or guide* or "Three-dimension*" or "Three dimension*" or 3D or "3-D")):ti,ab,kw |
| #9 | (EnSite* or Carto* or NavX* or RPM* or LocaLisa* or Affera* or IntellaNav* or "Rhythmia HDx*" or KODEX-EPD):ti,ab,kw |
| #10 | #5 OR #6 OR #7 OR #8 OR #9 |
| #11 | #4 AND #10 |

| CINAHL (EBSCOhost) | |
| --- | --- |
| S1 | (MH "Bundle of HIS") |
| S2 | TI ( ((His-bundle or His or Branch or "conduction system" or antybradycardia) N3 (pacing or bundle*)) ) OR AB ( ((His-bundle or His or Branch or "conduction system" or antybradycardia) N3 (pacing or bundle*)) ) OR SU ( ((His-bundle or His or Branch or "conduction system" or antybradycardia) N3 (pacing or bundle*)) ) |
| S3 | (TI ( ("His bundle pacing" or HBP or "Left bundle branch pacing" or LBBP or "Left bundle branch area pacing" or LBBAP) ) OR AB ( ("His bundle pacing" or HBP or "Left bundle branch pacing" or LBBP or "Left bundle branch area pacing" or LBBAP) ) OR SU ( ("His bundle pacing" or HBP or "Left bundle branch pacing" or LBBP or "Left bundle branch area pacing" or LBBAP) ) |
| S4 | S1 OR S2 OR S3 |
| S5 | TI ( (Electromapping or Electro-mapping or EAM or EAM-guided or 3D-EAM) ) OR AB ( (Electromapping or Electro-mapping or EAM or EAM-guided or 3D-EAM) ) OR SU ( (Electromapping or Electro-mapping or EAM or EAM-guided or 3D-EAM) ) |
| S6 | TI ( ((Intracardiac or intra-cardiac or "nonfluoroscop*" or "without fluoroscop*" or "zero-fluoroscop*" or "near-zero fluoroscop*" or "zero fluoroscop*" or "zero x-ray" or "alternative fluoroscop*" or "fluoroscopy elimination" or "radiation-free" or "fluoroless" or "fluoroscop*-free or non-fluoroscop*) N5 (navigat* or intervention or electroanatom* or electro-anatom* or electrogram*)) ) OR AB ( ((Intracardiac or intra-cardiac or "nonfluoroscop*" or "without fluoroscop*" or "zero-fluoroscop*" or "near-zero fluoroscop*" or "zero fluoroscop*" or "zero x-ray" or "alternative fluoroscop*" or "fluoroscopy elimination" or "radiation-free" or "fluoroless" or "fluoroscop*-free or non-fluoroscop*) N5 (navigat* or intervention or electroanatom* or electro-anatom* or electrogram*)) ) OR SU ( ((Intracardiac or intra-cardiac or "nonfluoroscop*" or "without fluoroscop*" or "zero-fluoroscop*" or "near-zero fluoroscop*" or "zero fluoroscop*" or "zero x-ray" or "alternative fluoroscop*" or "fluoroscopy elimination" or "radiation-free" or "fluoroless" or "fluoroscop*-free or non-fluoroscop*) N5 (navigat* or intervention or electroanatom* or electro-anatom* or electrogram*)) ) |
| S7 | TI ( ((electroanatom* or electro-anatom* or electrogram* or system* or navigat* or tecnique*) N3 (mapping* or mapping-guide* or guide* or Three-dimension* or "Three dimension*" or 3D or 3-D)) ) OR AB ( ((electroanatom* or electro-anatom* or electrogram* or system* or navigat* or tecnique*) N3 (mapping* or mapping-guide* or guide* or Three-dimension* or "Three dimension*" or 3D or 3-D)) ) OR SU ( ((electroanatom* or electro-anatom* or electrogram* or system* or navigat* or tecnique*) N3 (mapping* or mapping-guide* or guide* or Three-dimension* or "Three dimension*" or 3D or 3-D)) ) |
| S8 | TI (EnSite* or "Carto system*" or NavX* or RPM* or LocaLisa* or Affera* or IntellaNav* or "Rhythmia HDx*" or KODEX-EPD ) OR AB (EnSite* or Carto system* or NavX* or RPM* or LocaLisa* or Affera* or IntellaNav* or Rhythmia HDx* or KODEX-EPD ) OR SU (EnSite* or Carto system* or NavX* or RPM* or LocaLisa* or Affera* or IntellaNav* or Rhythmia HDx* or KODEX-EPD ) |
| S9 | SU "device implantation" OR AB ( EnSite* or Carto system* or NavX* or RPM* or LocaLisa* or Affera* or IntellaNav* or Rhythmia HDx* or KODEX-EPD ) OR SU ( EnSite* or Carto system* or NavX* or RPM* or LocaLisa* or Affera* or IntellaNav* or Rhythmia HDx* or KODEX-EPD ) |
| S10 | (S5 OR S6 OR S7 OR S8 OR S9) |
| S11 | (S4 AND S10) |

Table S2.1. Parameters for estimating the equivalent cost per minute of the X-ray tube for the administration of fluoroscopy

| Parameter | Value [Source] |
| --- | --- |
| Resale value (S) | 0 |
| Years of useful life (n) | 7 (27) |
| Interest rate (r) | 0.03 (25) |
| Annuity factor A (n,r) | 0.81 |
| Acquisition price (K) (€, without VAT) | 30 000 [Experts] |
| Equivalent annual cost (E) (€, without VAT) | 4815.19 |
| No. of target patients per year | 207 [Estimated] |
| Average duration of intervention (minutes per session) * | 119.77 [SR of effectiveness and safety] |
| * The average implantation time with fluoroscopy was assumed, given that there are no significant differences between the total duration of both types of interventions. | |

Table S2.2. Parameters for estimating the equivalent cost per patient of the Ensite navigator

| Parameter | Value [Source] |
| --- | --- |
| Resale value (S) | 0 |
| Years of useful life (n) | 5 [Abbott] |
| Interest rate (r) | 0.03 (25) |
| Annuity factor A (n,r) | 4.58 |
| Acquisition price (K) (€, without VAT) | 170 000 [Abbott] |
| Equivalent annual cost (E) (€, without VAT) | 37 120.28 |
| No. of target patients per year | 207 [Estimated] |

Table S3. Studies excluded in the review of effectiveness and safety

| Excluded by design / type of publication |
| --- |
| 1. Acosta H, Acosta NJ, Lopera G. Representation of the His Bundle Cloud on a Three-dimensional Electroanatomical Map and Its Implications for His Bundle Pacing. J Innov Card Rhythm Manag. 2021 may;12(5):4498-9. |
| 1. Burri H, Jastrzebski M, Cano Ó, Čurila K, de Pooter J, Huang W, et al. EHRA clinical consensus statement on conduction system pacing implantation: executive summary. Endorsed by the Asia-Pacific Heart Rhythm Society (APHRS), Canadian Heart Rhythm Society (CHRS) and Latin-American Heart Rhythm Society (LAHRS). Europace. 2023 abr 15;25(4):1237-48. |
| 1. Cay S, Ozeke O, Ozcan F, Topaloglu S, Aras D. Simple Approaches to Reduce Radiation in the Electrophysiology Laboratory. 2020 |
| 1. Coluccia G, Accogli M, Guido A, Chiarillo MV, Panico V, Sergi C, et al. Concomitant His bundle pacing and atrioventricular junction ablation: feasibility of a three-dimensional mapping system-only-guided, zero-fluoroscopy approach. J Cardiovasc Med (Hagerstown). 2021 dic 1;22(12):e21-3. |
| 1. Coluccia G, Accogli M, Panico V, Sergi C, Guido A, Palmisano P. Is it feasible to perform permanent left bundle branch area pacing, guided only by an electroanatomical mapping system? Proposal of a zero-fluoroscopy approach. HeartRhythm Case Rep. 2022 ene 1;8(4):233-7. |
| 1. Ge X, Chen M, Sha Z, Zhang J. Three-dimensional mapping in cardiac implantable electronic device - a feasible and effective alternative to fluoroscopy. J Interv Card Electrophysiol. 2023 abr;66(3):783-92. |
| 1. Hua W, Gu M, Niu H, Gold MR. Advances of Implantation Techniques for Conduction System Pacing. JACC Clin Electrophysiol. 2022 dic;8(12):1587-98. |
| 1. Hua W, Hu Y, Gu M, Cai C, Chen X, Niu H, et al. A feasible approach for His bundle pacing using a novel mapping system in patients receiving pacemaker therapy. HeartRhythm Case Rep. 2019 jun 21;5(8):433-5 |
| 1. Ponnusamy SS, Vijayaraman P. Electroanatomical mapping assisted conduction system pacing. Indian Pacing Electrophysiol J. 2022 jun 28;22(4):186-7. |
| 1. Ramos-Maqueda J, Alarcón F, Cabrera-Ramos M. Zero fluoroscopy approach for cardiac resynchronization therapy using left bundle branch area pacing. J Interv Card Electrophysiol. 2022 nov;65(2):327-8. |
| 1. Sharma PS, Huang HD, Trohman RG, Naperkowski A, Ellenbogen KA, Vijayaraman P. Low Fluoroscopy Permanent His Bundle Pacing Using Electroanatomic Mapping: A Feasibility Study. Circ Arrhythm Electrophysiol. 2019 feb;12(2):e006967. |

Table S4.1. Meta-analysis summary

| **Outcome measure/subgroup** | **Studies** | **N** | **Statistical method** | **Estimated effect [95% CI]** | **Heterogeneity** |
| --- | --- | --- | --- | --- | --- |
| Successful procedure | 5 | 194 | RR | 0.98 [0.92 to 1.05] P= 0.59 | Chi^2^= 1.91, df= 4 (P= 0.75); I^2^= 0% |
| **Procedure details (implantation)** | | | | | |
| Procedural time (min) | 7 | 231 | MD | -2.66 [-16.13 to 10.81] P= 0.70 | Chi^2^= 26.05, df= 6 (P=0.0002); I^2^= 77% |
| Total fluoroscopy time (min) | 7 | 231 | MD | -9.87 [-14.20 to -5.53] P<0.00001 | Chi^2^= 129.08, df= 6 (P<0.00001); I^2^= 95% |
| Total fluoroscopy dose (mGy) | 3 | 80 | MD | -55.28 [-118.67 to 8.12] P= 0.09 | Chi^2^= 63.28, df= 2 (P<0.00001); I^2^= 97% |
| Paced QRS duration (ms) | 4 | 144 | MD | -3.92 [-9.43 to 1.60] P= 0.16 | Chi^2^= 1.97, df= 3 (P= 0.58); I^2^= 0% |
| His-ventricular interval duration (ms) | 2 | 104 | MD | -1.04 [-7.19 to 5.12] P= 0.74 | Chi^2^= 0.19, df= 1 (P= 0.67); I^2^= 0% |
| His lead fluoroscopy time (min) | 2 | 50 | MD | -8.08 [-10.36 to -5.81] P<0.00001 | Chi^2^= 0.07, df= 1 (P= 0.79); I^2^= 0% |
| His lead fluoroscopy dose (mGy) | 1 | 20 | MD | -17.21 [-24.08 to -10.34] P<0.00001 | No aplicable |
| **Stimulation parameters (implantation)** | | | | | |
| Capture threshold (V/1 ms) | 6 | 214 | MD | -0.02 [-0.15 to 0.12] P= 0.82 | Chi^2^= 13.41, df= 5 (P= 0.02); I^2^= 63% |
| Impedance (Ʊ) | 5 | 184 | MD | 16.51 [-15.28 to 48.30] P= 0.31 | Chi^2^= 4.79, df= 4 (P= 0.31); I^2^= 16% |
| R-wave amplitude (mV) | 6 | 214 | MD | 0.46 [-0.15 to 1.07] P= 0.14 | Chi^2^= 1.82, df= 5 (P= 0.87); I^2^= 0% |
| **Stimulation parameters (follow-up 1-6 months)** | | | | | |
| Capture threshold (V/1 ms) | 4 | 136 | MD | -0.08 [-0.23 to 0.07] P= 0.32 | Chi^2^= 3.95, df= 3 (P= 0.27); I^2^= 24% |
| Impedance (Ʊ) | 2 | 60 | MD | -7.31 [-50.17 to 35.55] P= 0.74 | Chi^2^= 0.02, df= 1 (P= 0.90); I^2^= 0% |
| R-wave amplitude (mV) | 3 | 90 | MD | -0.26 [-1.16 to 0.65] P= 0.58 | Chi^2^= 0.34, df= 2 (P= 0.84); I^2^= 0% |
| **Echocardiography** | | | | | |
| LVEF (%) (baseline) | 3 | 144 | MD | 0.14 [-3.43 to 3.70] P= 0.94 | Chi^2^= 0.67, df= 2 (P= 0.72); I^2^= 0% |
| LVEF (%) (3-6 months) | 2 | 60 | MD | 0.44 [-3.85 to 4.74] P= 0.84 | Chi^2^= 2.31, df= 1 (P= 0.13); I^2^= 57% |
| **Safety outcomes** | | | | | |
| Procedure-related complications (implantation) | 5 | 184 | RR | Not estimable | Not applicable |
| Lead displacement (1-6 months) | 4 | 174 | RR | 0.33 [0.01 to 7.86] P= 0.50 | Not applicable |
| Lead revision (1-6 months) | 1 | 46 | RR | Not estimable | Not applicable |
| Pneumothorax (1 month) | 1 | 30 | RR | Not estimable | Not applicable |
| Pericardial effusion (1 month) | 1 | 30 | RR | Not estimable | Not applicable |
| Increase in capture threshold (1 month) | 1 | 30 | RR | 0.64 [0.03 to 14.36] P= 0.78 | Not applicable |
| Device infection (1 month) | 1 | 30 | RR | Not estimable | Not applicable |
| MD: mean difference; CI: confidence interval; LVEF, left ventricular ejection fraction; RR: risk ratio | | | | | |

Figure S4.1. Forest plot of His lead fluoroscopy time (minutes)


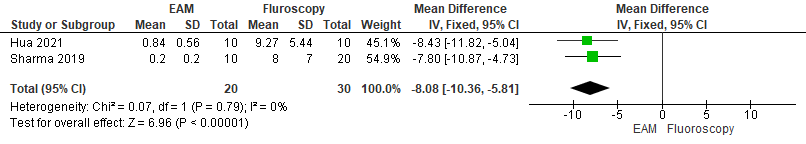


Figure S4.2. Forest plot of total fluoroscopy dose (mGy)


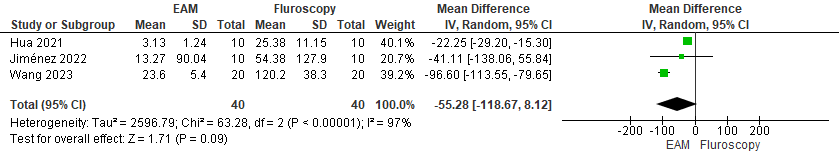


Figure S4.3. Forest plot of His Lead fluoroscopy dose (mGy)


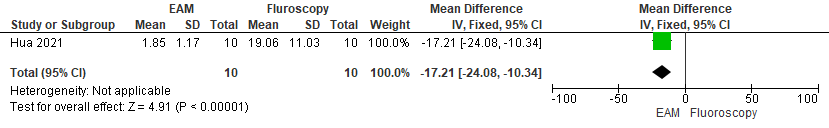


Figure S4.4. Forest plot of paced QRS duration (milliseconds)


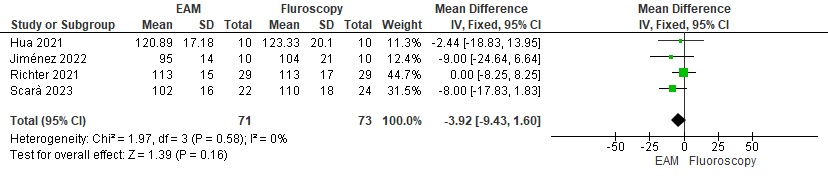


Figure S4.5. Forest plot of His-ventricular interval duration (milliseconds)


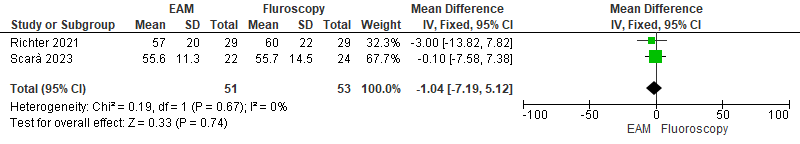


Figure S4.6. Forest plot of capture threshold (V/1 ms)


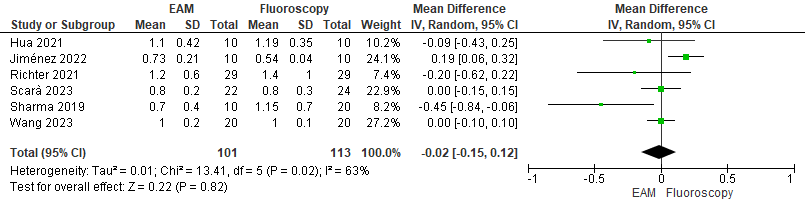


Figure S4.7. Forest plot of impedance (Ω)


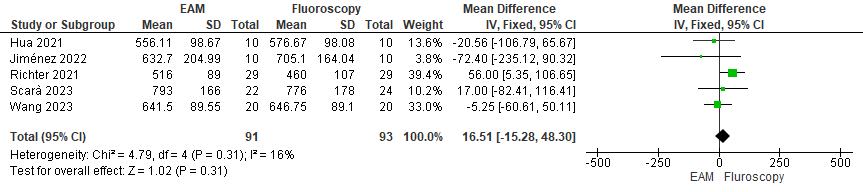


Figure S4.8. Forest plot of R-wave amplitude (mV)


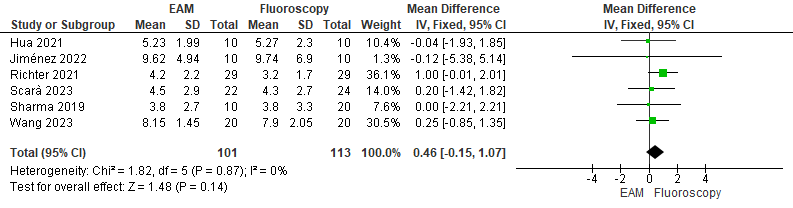


Figure S4.9 Forest plot of capture threshold (V/1 ms) during follow-up (1-6 months)


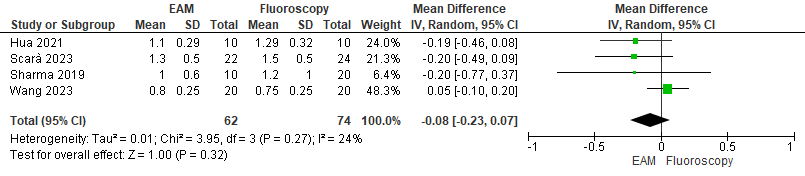


Figure S4.10. Forest plot of impedance (Ω) during follow-up (3-6 months)


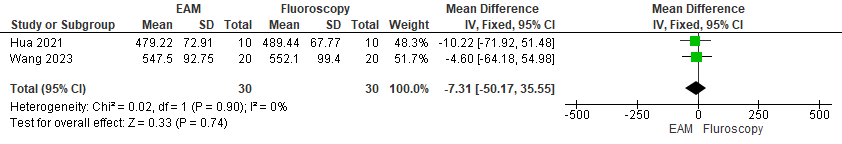


Figure S4.11. Forest plot of R-wave amplitude (mV) during follow-up (1-6 months)


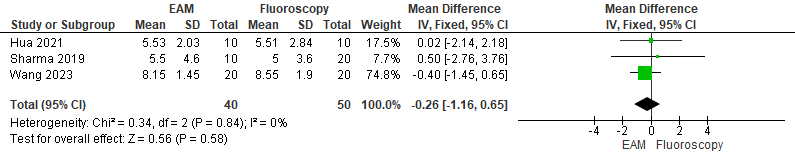


Figure S4.12. Forest plot of left ventricular ejection faction (%) during follow-up (3-6 months)


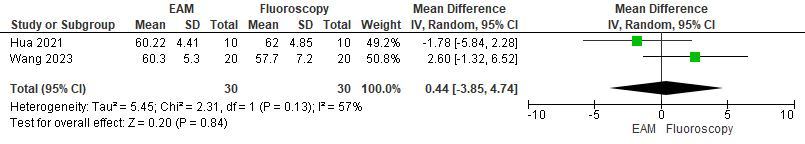


Table S5. Evidence profile

Question: Should electroanatomical mapping-guided system be used versus fluoroscopy for conduction system pacing in patients with bradyarrhythmias (symptomatic sinus node dysfunction or atrioventricular conduction blocks)?

| Table S5.1. GRADE evidence profile | | | | | | | | | | | | |
| --- | --- | --- | --- | --- | --- | --- | --- | --- | --- | --- | --- | --- |
| **Certainty assessment** | | | | | | | **Number of patients** | | **Effect** | | **Certainty** | **Significance** |
| Number of studies | Study design | Risk of bias | Inconsistency | Indirect evidence | Imprecision | Other considerations | EAM | Fluoroscopy | Relative (95% CI) | Absolute (95% CI) |  |  |
| **Successful procedure (implantation)** | | | | | | | | | | | | |
| 5^1,2,3,4,5^ | Observational studies | Serious^a^ | Not serious | Not serious | Not serious | None | 87/91 (95.6%) | 100/103 (97.1%) | **RR 0.98** (0.92 to 1.05) | **19 less per 1000** (78 less to 49 more) | ⨁◯◯◯ Very low | CRITICAL |
| **Total fluoroscopy dose (implantation, mGy)** | | | | | | | | | | | | |
| 3^4,5,6^ | Observational studies | Serious^j^ | Very serious^k^ | Not serious | Serious^l^ | None | 40 | 40 | - | MD **55.28 mGy** less (118.67 less to 8.12 more) | ⨁◯◯◯ Very low | IMPORTANT |
| **Procedure-related complications (implantation)** | | | | | | | | | | | | |
| 5^1,2,4,5,6^ | Observational studies | Serious^b^ | Not serious | Not serious | Not serious^c^ | None | 0/91 (0.0%) | 0/93 (0.0%) | Not estimable |  | ⨁◯◯◯ Very low | CRITICAL |
| **Lead displacement (follow-up: range 1 month to 6 months)** | | | | | | | | | | | | |
| 4^1,2,3,4^ | Observational studies | Serious^d^ | Not serious^e^ | Not serious | Serious^f^ | None | 0/81 (0.0%) | 1/93 (1.1%) | **RR 0.33** (0.01 to 7.86) | **7 less per 1000**  (11 less to 74 more) | ⨁◯◯◯ Very low | CRITICAL |
| **Lead revision (follow-up: average 6 months)** | | | | | | | | | | | | |
| 1^2^ | Observational studies | Serious^g^ | Not serious^h^ | Not serious | Not serious^c^ | None | 0/22 (0.0%) | 0/24 (0.0%) | Not estimable |  | ⨁◯◯◯ Very low | CRITICAL |
| **Device infection (follow-up: average 1 month)** | | | | | | | | | | | | |
| 1^3^ | Observational studies | Serious^i^ | Not serious ^h^ | Not serious | Not serious ^c^ | None | 0/10 (0.0%) | 0/20 (0.0%) | Not estimable |  | ⨁◯◯◯ Very low | CRITICAL |
| **Quality of life** | | | | | | | | | | | | |
| No studies were identified that evaluated this outcome. | | | | | | | | | | | | |
| **Mortality** | | | | | | | | | | | | |
| No studies were identified that evaluated this outcome. | | | | | | | | | | | | |
| CI: Confidence interval; EAM: Electroanatomical mapping-guided system; MD: Mean difference; RR: Risk ratio  **Explanations**  a. Four of the included studies presented a moderate risk of bias and one had a serious risk of bias.  b. Three of the included studies presented a moderate risk of bias, one a serious risk of bias, and another a critical risk of bias.  c. Imprecision could not be assessed because the effect could not be estimated.  d. Three of the included studies presented a moderate risk of bias and one had a serious risk of bias.  e. Heterogeneity not applicable because the effect could not be calculated for three of the four studies.  f. Imprecision was defined by wide confidence intervals. The CI includes the null effect and/or risk/benefit values (0.8 and 1.25).  g. The included study presented a serious risk of bias.  h. Heterogeneity not applicable because it is a single study.  i. The included study presented a moderate risk of bias.  j. Two of the included studies presented a moderate risk of bias and one had a critical risk of bias.  k. Heterogeneity of 97% (p<0.001).  l. Wide confidence interval.  **References**  1.Richter, S, Ebert, M, Bertagnolli, L, Gebauer, R, Lucas, J, Scheller, D, Paetsch, I, Hindricks, G, Döring, M. Impact of electroanatomical mapping-guided lead implantation on procedural outcome of His bundle pacing. Europace; 2021 Mar 8.  2.Scarà, A, Golia, P, Grieco, D, Borrelli, A, De, Ruvo,E, Bressi, E, Politano, A, De, Luca,L, Bruni, G, Fagagnini, A, Panuccio, M, Rebecchi, M, Zecchi, P, Solimene, F, Calò, L, Sciarra, L. Low fluoroscopy permanent His bundle pacing using a new electroanatomic mapping system (KODEX EPD). A multicenter experience. J Arrhythm; 2022 Dec 30.  3.Sharma, PS, Huang, HD, Trohman, RG, Naperkowski, A, Ellenbogen, KA, Vijayaraman, P. Low Fluoroscopy Permanent His Bundle Pacing Using Electroanatomic Mapping: A Feasibility Study. Circ Arrhythm Electrophysiol; 2019 Feb.  4.Wang, L, Yang, S, Tang, B, Wang, F, Sang, W, Han, Y, Wang, L, Zhou, X, Zhang, J, Xing, Q, Tuerhong, Z, Xiaokereti, J, Guo, Y, Li, Y. Feasibility, safety and effectiveness of mapping system assisted conduction system pacing: a single-center prospective study. Sci Rep; 2023 Jun 15.  5.Hua, W, Liu, X, Gu, M, Niu, HX, Chen, X, Tang, M, Zhang, S. Novel Wide-Band Dielectric Imaging System Guided Lead Deployment for His Bundle Pacing: A Feasibility Study. Front Cardiovasc Med; 2021 Sep 3.  6.Jimenez, E, Gordon, A, Cortez, D. Reduction of fluoroscopy in conduction system pacing guided by electroanatomical mapping in pediatrics and congenital heart disease. Indian Pacing Electrophysiol J; 2022 Jul-Aug. | | | | | | | | | | | | |
